# Awareness of Preeclampsia and Its Associated Factors Among Women in Al Baha Region, Saudi Arabia

**DOI:** 10.7759/cureus.49038

**Published:** 2023-11-19

**Authors:** Tajelsir Osman, Eman A Keshk, Abdullah Ali S Alghamdi, Mohammed Ahmed A Alghamdi, Mohammed Abdullah A Alghamdi, Ahmed A Alzahrani, Khalid N Alghamdi, Yasser A Alzahrani, Abdulrahman A Alghamdi, Rahaf A Alghamdi

**Affiliations:** 1 Obstetrics & Gynecology, Al Baha University, Al Baha, SAU; 2 College of Medicine, Al Baha University, Al Baha, SAU

**Keywords:** risk factor, awarness, ob-gyn, pregnancy-related complications, obesity, pulmonary function, hypertension, high-risk pregnancy, obs&gy, pre-eclampsia

## Abstract

Background: Preeclampsia is associated with the incidence of common fetal problems including intra-uterine growth restriction (IUGR), premature delivery oligohydramnios, placental abruption, fetal discomfort, and intrauterine fetal death. Pregnant women are not well-informed about preeclampsia, including its symptoms, risk factors, and consequences. The aim of the current study is to evaluate the awareness of preeclampsia and its associated factors among women in the Al Baha region, Saudi Arabia.

Methodology: An observational cross-sectional design was employed to assess the awareness of preeclampsia and its associated factors among women in the Al Baha region of Saudi Arabia. Data was collected from April 2023 to September 2023. A questionnaire was designed to gather information on participants' sociodemographic characteristics (such as age, educational level, and residency) and their awareness of preeclampsia, including knowledge about signs/symptoms, risk factors, and complications.

Results: In the current study, we included 485 pregnant women. The majority of participants were aged 40 years or older (37.5%), followed by those aged 35-39 (20.4%). Among the participants, 70.9% confirmed that they had heard about pre-eclampsia before. The most common signs and symptoms were high blood pressure (47.4%), increased protein in urine (40.2%), continuous headache (39.2%), and vomiting/nausea (40.0%). Participants demonstrated awareness of obesity (29.7%), diabetes mellitus (35.5%), chronic hypertension (47.0%), and chronic kidney disease (31.3%) as major risk factors. Participants were aware of potential risks such as kidney disorders (34.6%), heart disorders (23.7%), and preterm delivery (50.9%). The analysis reveals that younger participants below 20 years old (3.3%) and lower educational levels (5.6%) had lower awareness of preeclampsia compared to older age groups.

Conclusion: The findings of this study highlight a reasonable level of preeclampsia awareness and knowledge among Saudi Arabian women residing in the Al Baha region. While the majority of participants were familiar with preeclampsia, there were significant knowledge gaps regarding the precise symptoms, risk factors, and consequences of the condition

## Introduction

Hypertensive disorders of pregnancy occur in 10% of pregnant women [1]. This condition is defined by the International Society for the Study of Hypertension in Pregnancy (ISSHP) as hypertension (140 mmHg systolic or 90 mmHg diastolic) after 20 weeks of gestation [2]. Complications for the mother and the fetus are linked to it. Long-term hypertension and stroke risk are increased for the mother [3]. Common fetal problems include intra-uterine growth restriction (IUGR), premature delivery oligohydramnios, placental abruption, fetal discomfort, and intrauterine fetal death [4].

Predicting whether women are likely to develop preeclampsia (PE) during antenatal visits is a crucial step. All women should understand the warning signs and symptoms of PE. According to the 2019 National Center for Health and Care Excellence (NICE) guidelines, a woman is considered to be at high risk of PE if she has a history of hypertensive disease during a prior pregnancy or maternal conditions like chronic renal failure, autoimmune diseases, diabetes, or chronic hypertension [3]. If a woman is nulliparous, younger than 40, has a body mass index (BMI) below 35 kg/m^2^ [2], has a family history of PE, is carrying more than one fetus, or has a pregnancy interval longer than 10 years [3], she is at intermediate risk.

PE is a multisystem illness linked to pregnancy that is believed to have two stages. The first stage includes the impairment of local placental hypoxia and fetal trophoblastic invasion of the decidua [5]. The second stage involves abnormal production of proinflammatory, antiangiogenic, and angiogenic factors as well as the release of placental blood-related substances into the maternal circulation [6]. Women with PE also exhibit a variety of indications and symptoms that are related to various organ systems. The multi-organ system dysfunction in PE frequently results in headaches, visual abnormalities, abnormal renal function, severe hypertension, chest discomfort, pulmonary edema and low oxygen saturation, nausea, and abnormal liver function, among other symptoms [7,8]. In the current study, we seek to gauge the understanding of PE by women in Al Baha, Saudi Arabia, and its contributing factors because prior research in the United States [9] and a few African nations [10-12] suggests that knowledge of PE among women is generally low.

## Materials and methods

Study design

An observational cross-sectional design was employed to assess the awareness of PE and its associated factors among women in the Al Baha region of Saudi Arabia. The study aimed to gather data from April 2023 to December 2023, spanning a period of nine months. Ethical approval was given by the Scientific Research and Ethical Committee, Faculty of Medicine, Al Baha University, Saudi Arabia (approval number: REC/OB/BU-FM/2023/17 dated May 21, 2023). All participants gave consent before starting the questionnaire. No personal data of the participants were used and all data were saved confidentially.

Study population and sampling

The study population consisted of resident women in the Al Baha region of Saudi Arabia who were of reproductive age (18-49 years), had at least one previous pregnancy, and were able to read and understand Arabic. Participants were required to have a social media account to access and complete the online questionnaire. Exclusion criteria included women who were not willing to participate in the study, had previously been diagnosed with PE (described in Arabic as “تسمم الحمل (Tasmm el haml)” to ensure that all participants understand the term), or working as healthcare providers.

Simple random sampling was used to select participants from the population. The sample size was determined using the Epi Info™ program (Centers for Disease Control and Prevention, Atlanta, Georgia, United States), considering a 95% confidence interval, a 5% margin of error, and the total population of Al Baha. The estimated sample size was 384, adjusted to 422 to account for a 10% non-response rate. The sampling frame was obtained from the Al Baha Regional Health Administration, which provided a list of women residing in the region.

Data collection and instrument

Data was collected through a Google Form (Google LLC, Mountain View, California, United States) questionnaire that was administrated in Arabic. The questionnaire was developed by researchers based on relevant literature and existing validated scales. It consisted of several sections to gather information on participants' sociodemographic characteristics, including age, educational level, residency, and work status. The questionnaire also assessed participants' awareness of PE, including knowledge about signs/symptoms, risk factors, and complications. The items from the questionnaire were designed to be clear and concise to ensure accurate responses. A pilot study was conducted with 20 participants to assess the clarity and suitability of the questionnaire and estimate the time required for data collection. The pilot study participants were excluded from the final analysis.

Data analysis

The collected data were coded and entered into IBM SPSS Statistics for Windows, Version 26.0 (Released 2019; IBM Corp., Armonk, New York, United States) for statistical analysis. Descriptive statistics, such as frequencies and percentages, were used to summarize the sociodemographic characteristics of the participants. Chi-square tests were employed to examine the association between sociodemographic factors and PE awareness. The normality of the data was assessed using a combination of statistical tests and graphical methods in SPSS. Student t-tests were used to compare means between groups, and Spearman's correlation coefficient was calculated to assess the relationship between variables. The significance level was set at p < 0.05.

## Results

Table 1 presents the demographic factors of the study participants. The majority of participants were aged 40 years or older (37.5%), followed by those aged 35-39 (20.4%). A significant proportion fell within the 25-29 and 30-34 age groups, accounting for 14.4% and 14.2% of the participants, respectively. The youngest age group (≤ 20 years) comprised 3.3% of the participants. Regarding educational level, the majority had a university education (75.1%), while high school education was reported by 19.4% of participants. A smaller percentage had intermediate school level education (2.5%), and the lowest proportion had a primary school education (3.1%). In terms of occupation, the most common category was teachers (33.6%), followed by housewives (23.7%) and those not working (16.1%). Students accounted for 5.4% of the participants, while other occupations, such as health-related jobs and private business, were reported by smaller percentages. Regarding the number of pregnancies, the majority of participants (55.3%) reported having two to five pregnancies. Approximately a quarter of participants (25.6%) reported having only one pregnancy, while 19.2% reported having more than five pregnancies.

**Table 1:** Demographic factors of the participants

Variables		Frequency	Percentage
Age (years)	< 20	16	3.3%
	20-24	49	10.1%
	25-29	70	14.4%
	30-34	69	14.2%
	35-39	99	20.4%
	40 or more	182	37.5%
Educational level	Primary school	15	3.1%
	Intermediate school	12	2.5%
	High school	94	19.4%
	University	364	75.1%
Work	Not working	78	16.1%
	Student	26	5.4%
	Housewife	115	23.7%
	Teacher	163	33.6%
	Educational supervisor	9	1.9%
	Health-related jobs	23	4.7%
	Private works	10	2.1%
	Other	61	12.6%
Number of pregnancies:	1	124	25.6%
	2-5	268	55.3%
	more than 5	93	19.2%

Table 2 presents the prevalence of hypertension and PE during pregnancy among the participants. Regarding the diagnosis of high blood pressure during previous pregnancies, 17.5% of participants confirmed having been diagnosed, while 4.5% were unsure. In terms of PE diagnosis during previous pregnancies, 7.8% of participants confirmed having been diagnosed, while 4.1% were unsure. Among those diagnosed with high blood pressure during pregnancy, 24.1% confirmed having excess protein in their urine. When asked if their doctors had informed them of having PE when diagnosed with high blood pressure during pregnancy, 22.9% of participants confirmed being informed, and 16.8% were unsure. Regarding the experience of high blood pressure during other pregnancies, 25.4% of participants confirmed experiencing it. In terms of cesarean section births, 41.6% of participants confirmed having had one. Of the respondents, 8.0% had a gestational age below 34 weeks, 47.0% had a gestational age between 34-37 weeks, and 44.9% had a gestational age above 37 weeks. When asked if they currently suffer from high blood pressure, 12.6% of participants confirmed having it, and 7.0% were unsure. Among those with high blood pressure, 11.8% reported being prescribed blood pressure-lowering medications. Regarding family history of PE, 44.1% of participants confirmed having a family history. Among those with a family history, the majority reported the relative's relation as parents (33.0%).

**Table 2:** Prevalence of hypertension and preeclampsia during pregnancy

Variables		Frequency	Percentage
Have you been exclusively diagnosed with high blood pressure during previous pregnancies?	No	378	77.9%
	Yes	85	17.5%
	I do not know	22	4.5%
Have you been diagnosed with high blood pressure during previous pregnancies?	No	427	88.0%
	Yes	38	7.8%
	I do not know	20	4.1%
When you were diagnosed with high blood pressure during pregnancy, did you have excess protein in your urine?	No	57	42.9%
	Yes	32	24.1%
	I do not know	44	33.1%
When you were diagnosed with high blood pressure during pregnancy, did your doctor tell you that you had pre-eclampsia?	No	79	60.3%
	Yes	30	22.9%
	I do not know	22	16.8%
Did you suffer from high blood pressure during other pregnancies as well?	No	100	74.6%
	Yes	34	25.4%
Was one of your births a cesarean section?	No	283	58.4%
	Yes	202	41.6%
How old was your gestational fetus (in weeks) when it was born?	< 34 weeks	39	8.0%
	34-37 weeks	228	47.0%
	> 37 weeks	218	44.9%
Have you been hypnotized before during pregnancy?	No	206	42.5%
	Yes, for childbirth only	187	38.6%
	Yes, because of the premature birth	43	8.9%
	Yes, due to high blood pressure or pre-eclampsia	29	6.0%
	Gestational diabetes	7	1.4%
	Other	13	2.7%
Do you suffer from high blood pressure now?	No	390	80.4%
	Yes, started after pregnancy	41	8.4%
	Yes, continued after pregnancy	21	4.2%
	I do not know	34	7.0%
Are you currently prescribed blood pressure-lowering medications?	No	428	88.2%
	Yes	57	11.8%
Family history of preeclampsia	No	271	55.9%
	Yes	214	44.1%
Relative's relation	No	271	55.9%
	Parents	160	33.0%
	Siblings	32	6.6%
	Father/mother brother or sister	9	1.9%
	Grandparent	13	2.7%

**Figure 1:**
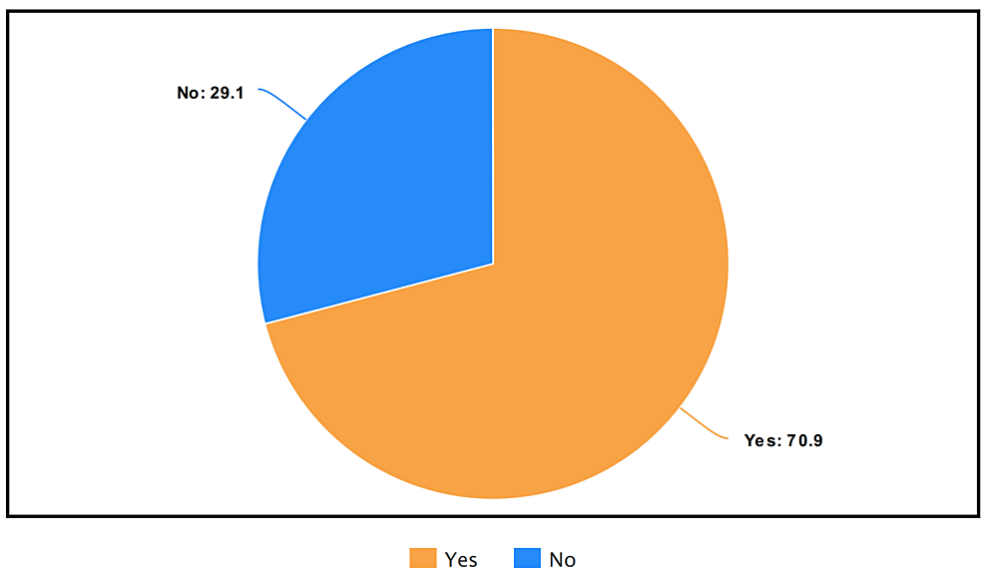
Response of participants when questioned whether they had heard about preeclampsia before

Among the participants, 70.9% confirmed that they had heard about high blood pressure in pregnancy before, while 29.1% reported not being familiar with it (Figure 1). Regarding the timing of PE during pregnancy, 63.1% of participants believed that a pregnant woman suffered from PE at 20 weeks, while 36.9% believed it to be within the first week of pregnancy.

Table 3 summarizes the participants' knowledge regarding symptoms, risk factors, and consequences of PE. The findings indicate that a significant proportion of participants were aware of key signs and symptoms such as hypertension (47.4%), increased protein in urine (40.2%), continuous headache (39.2%), and vomiting/nausea (40.0%). However, awareness was relatively lower for symptoms such as chest pain (17.7%) and vision disorders (22.3%). When it comes to risk factors, participants demonstrated awareness of obesity (29.7%), diabetes mellitus (35.5%), chronic hypertension (47.0%), and chronic kidney disease (31.3%). Knowledge about the risk associated with pregnancy with more than one fetus was relatively lower (22.5%). Regarding consequences, participants were aware of potential risks such as kidney disorders (34.6%), heart disorders (23.7%), and preterm delivery (50.9%). However, awareness of other consequences like death (41.2%) and delayed fetus development (36.5%) was relatively lower. It is worth noting that a significant majority of participants (56.9%) reported being aware of the dangers of PE and actively working to avoid it. 

**Table 3:** Knowledge of the participants about symptoms, risk factors, and consequences of preeclampsia

Variables		No		Yes		I do not know	
		Frequency	Percentage	Frequency	Percentage	Frequency	Percentage
Knowledge considering symptoms of preeclampsia	Hypertension	37	7.6%	230	47.4%	218	44.9%
	Increased protein in urine	32	6.6%	195	40.2%	258	53.2%
	Continuous headache	33	6.8%	190	39.2%	262	54.0%
	Vomiting/nausea	37	7.6%	194	40.0%	254	52.4%
	Chest pain	65	13.4%	86	17.7%	334	68.9%
	Abdominal pain	42	8.7%	155	32.0%	288	59.4%
	Backache	46	9.5%	121	24.9%	318	65.6%
	Vision disorders	63	13.0%	108	22.3%	314	64.7%
	Seizures	43	8.9%	130	26.8%	312	64.3%
Knowledge considering risk factors of preeclampsia	Obesity	80	16.5%	144	29.7%	261	53.8%
	Diabetes mellitus	59	12.2%	172	35.5%	254	52.4%
	Chronic hypertension	37	7.6%	228	47.0%	220	45.4%
	Chronic kidney disease	40	8.2%	152	31.3%	293	60.4%
	Pregnancy with more than one fetus in one pregnancy	81	16.7%	109	22.5%	295	60.8%
Knowledge considering consequences of preeclampsia	Death	53	10.9%	200	41.2%	232	47.8%
	Kidney disorder	34	7.0%	168	34.6%	283	58.4%
	Heart disorders	54	11.1%	115	23.7%	316	65.2%
	Fetus mortality	28	5.8%	254	52.4%	203	41.9%
	Delayed fetus development	36	7.4%	177	36.5%	272	56.1%
	Preterm delivery	23	4.7%	247	50.9%	215	44.3%
Do you realize how dangerous preeclampsia is and are you working to avoid it?		45	9.3%	276	56.9%	164	33.8%

The results from Table 4 demonstrate the relationship between awareness of PE and various demographic factors. The analysis reveals that younger participants (below 20 years old) had lower awareness of PE compared to older age groups. Education level also played a significant role, with participants who had a primary school education exhibiting lower awareness compared to those with higher education levels. However, there was no significant difference in awareness based on the number of pregnancies or a history of high blood pressure during previous pregnancies. Notably, participants who had been diagnosed with PE during previous pregnancies showed higher awareness compared to those without such a history. 

**Table 4:** The relation between awareness of preeclampsia and demographic factors *significant

Variables		Have you heard about preeclampsia?				
		No		Yes		p-value
		Frequency	Percentage	Frequency	Percentage	
Age (years)	< 20	9	56.3%	7	43.8%	0.003*
	20-24	18	36.7%	31	63.3%	
	25-29	27	38.6%	43	61.4%	
	30-34	20	29.0%	49	71.0%	
	35-39	31	31.3%	68	68.7%	
	40 or more	36	19.8%	146	80.2%	
Educational level	Primary school	8	53.3%	7	46.7%	0.001*
	Intermediate school	5	41.7%	7	58.3%	
	High school	39	41.5%	55	58.5%	
	University	89	24.5%	275	75.5%	
Number of pregnancies:	1	45	36.3%	79	63.7%	0.094
	2-5	74	27.6%	194	72.4%	
	more than 5	22	23.7%	71	76.3%	
Have you been diagnosed with high blood pressure during previous pregnancies?	No	107	28.3%	271	71.7%	0.084
	Yes	23	27.1%	62	72.9%	
	I do not know	11	50.0%	11	50.0%	
Have you been diagnosed with pre-eclampsia during previous pregnancies?	No	123	28.8%	304	71.2%	0.002*
	Yes	6	15.8%	32	84.2%	
	I do not know	12	60.0%	8	40.0%	

## Discussion

The findings of this study shed light on the level of PE awareness and knowledge among Saudi Arabian women residing in the Al Baha region. PE is a serious pregnancy complication [13-15], and increasing knowledge and education about this illness are crucial for improving mother and fetal outcomes.

PE awareness and knowledge are moderate among the study population, according to the findings. The majority of responders (70.9%) stated that they were familiar with PE. This is contrary to a previous study conducted among pregnant women in the Makkah region of Saudi Arabia, in which the authors reported a high prevalence of insufficient PE knowledge among the study population (96%) [16]. Other studies by Savage and Hoho [11] and Eze et al. [10] also found that 59% and 60% of Tanzanian women, respectively, had inadequate knowledge of PE. Evidence suggests that a sufficient understanding of an illness contributes to its prevention, control, and management because patient knowledge of a disease positively influences patient adherence to treatment and aids in reducing disease-related consequences [17,18]. This suggests that the majority of women in the region of the current study have been exposed to information regarding this illness. However, there were considerable knowledge and awareness gaps regarding the precise symptoms, risk factors, and consequences of PE.

In terms of symptoms, almost half of the women were aware of the major indicators, such as hypertension, proteinuria, headaches, and nausea/vomiting. However, understanding of other key symptoms, such as vision problems and chest trouble, was far lower. This is in parallel with a previous study which showed that about half of the participants identified high blood pressure as one of the symptoms/signs of PE, while other specific symptoms, such as persistent headache, blurred vision, chest pain, nausea/vomiting, and convulsions, were less likely to be identified or recognized [16] and a study conducted in Tanzania, which found that knowledge about signs and symptoms of PE is very low [11]. This is concerning since failure to notice these symptoms could delay diagnosis and treatment. Similarly, while awareness of common risk factors such as obesity, diabetes, and chronic hypertension was relatively high, understanding of other significant concerns such as multiple gestation pregnancy was deficient. Similarly, the study in Makkah showed that less than half of the participating women recognized risk factors such as a family history of PE, thrombophilia, having a personal history of PE, and diabetes [16].

Variable degrees of knowledge on the potential maternal and fetal effects of PE were observed among the participants. Most were aware of dangers such as kidney disease, heart problems, and premature birth. However, awareness was lower for potentially deadly outcomes such as maternal mortality and decreased fetal growth. As PE has a high case fatality rate compared with other pregnancy-induced hypertensive disorders [13,19-21], this knowledge gap is worrisome and demonstrates the need for additional education.

Intriguingly, the study also investigated the association between PE awareness and various demographic characteristics. Younger women, those with a lower level of education, and residents of Al Baha city showed a lower degree of awareness than their counterparts. In a previous study by Gari et al., the authors found that a higher level of knowledge about PE is associated with the age group of 26-35 years and university education [16]. Similarly, a study by Mekie et al. conducted in Ethiopia concluded that a higher educational level contributes to better knowledge of PE [22]. This emphasizes the importance of focusing PE education efforts on vulnerable communities who presumably have less access to health information. Contrary to expectations, there was no association between parity and personal history of hypertension diseases and awareness. However, women with a history of PE possessed better knowledge, highlighting the importance of experiential learning, which is similar to previous studies [16,22].

In general, although the populace is aware of the symptoms, risk factors, and consequences of PE, there are significant knowledge gaps. Preventing, diagnosing, and treating PE early and effectively are hampered by a lack of knowledge about the complete spectrum of PE symptoms, predictors, and effects [23,24]. Certain sociodemographic characteristics, such as age, education, and location, are related to even lower levels of awareness, as was described previously.

The lack of PE knowledge among women in Al Baha is consistent with the findings of similar research conducted in Saudi Arabia and the region, highlighting the need for increased PE education. Participants' limited comprehension of PE may be attributable to a number of factors such as health literacy levels, access to quality antenatal care, sociocultural variables, and insufficient patient-provider communication are potential obstacles [25-27].

The study has several limitations that should be considered when interpreting the results. Firstly, the use of a cross-sectional design limits the establishment of causal relationships between variables, providing only a snapshot of PE awareness at a specific point in time. Secondly, the reliance on self-reported data through a questionnaire may introduce recall bias, as participants may not accurately recall or report their experiences and awareness levels. Additionally, the study's generalizability may be restricted to the Al Baha region of Saudi Arabia, and caution should be exercised when extrapolating the findings to other populations.

## Conclusions

The findings of this study highlight the moderate level of PE awareness and knowledge among Saudi Arabian women residing in the Al Baha region. While the majority of participants were familiar with PE, there were significant knowledge gaps regarding the precise symptoms, risk factors, and consequences of the condition. This lack of understanding poses a risk to the timely diagnosis and effective management of PE, which can have serious implications for both the mother and the fetus. By bridging the existing knowledge gaps and empowering women with the necessary information, healthcare providers and policymakers can work towards improving maternal and fetal health outcomes in the region. Antenatal care should include comprehensive teaching about PE. Additionally, patient-provider dialogue and community engagement can aid in closing knowledge gaps. Increasing awareness is essential for helping women recognize PE risks, seek treatment promptly, and improve maternal and fetal outcomes.
